# 

**Published:** 1896-04

**Authors:** 


					﻿CONTENTS
ORIGINAL ARTICLES—
Memorial of Dr. T. S. Powell. By Dr. J. McFadden Gaston,
Atlanta, Ga.....................................  157
Acetanilid as a Local Antiseptic and Analgesic. By Lucien
Lofton, M. D., Atlanta, Ga.....................   168
New York Letter .................................. 171
SELECTIONS AND ABSTRACTS—
The Surgical Treatment of -Retro-Deviations of the Uterus....	173
Penis Amputation. By Ramon Guiteras, M. D., New York.	175
Tuberculosis and Neoplasm of the Bladder ; Surgery or Hygiene? 176
Penetrating Wounds of the Chest................... 178
SOCIETY REPORTS—
Maryland Clinical Society.......................... 179
EDITORIAL—
Premature Burial..................................  189
The X Ray.......................................    190
Dr. Nathan O. Harris............................... 192
American Academy of Medicine........•.............. 196
SPECIAL NOTES.......................................   200
Prescriptions...................................... 208
				

